# High sensitive aptasensing chronic myeloid leukemia on circular electrode-modified by single-walled carbon nanotube

**DOI:** 10.1016/j.heliyon.2024.e36552

**Published:** 2024-08-20

**Authors:** Chuntao Zong, Xuebing Ran

**Affiliations:** a Department of Hematology, Xi'an Daxing Hospital, Xi'an, Shaanxi province, 710082, China; b Department of Clinical Laboratory, Meixian People's Hospital, Baoji City, 722300, China

**Keywords:** Blood cancer, Interdigitated electrode, Biomarker, Aptasensor, Nanomaterial

## Abstract

Chronic myeloid leukemia (CML) is a cancer in the bone marrow caused by the proliferation of granulocyte cells at all the maturation stages. Late diagnosis of CML decreases the patient survival rate, makes diagnosing CML is mandatory before entering the blastic phase. CD 19 is an important target for CML and is effectively utilized for therapeutic and diagnosis purposes. This research was focused on developing an aptamer-mediated circular interdigitated electrode (IDE) sensor for detecting the level of CD 19 and measured at 0–2 V with the step of 0.1 V. To improve the surface functionalization on IDE, the surface of IDE was modified with a single-walled carbon nanotube (SWCN) to enhance the aptamer immobilization. SWCN increased the aptamer attachment and also enhanced the analytical performances on IDE. This SWCN-aptamer modified IDE detected the CD 19 as low as 10 nM on a linear co-regression range from 10 to 100 nM [y = 2.0126x - 2.3857; R^2^ = 0.9749]. Furthermore, control performances with CD 33, and complementary aptamer did not show the increment of current, and CD 19 spiked human serum increased the current flow without significant interference, demonstrating the specific and selective detection of CD 19. This biosensor quantifies CD 19 biomarker at its lower level and diagnoses CML and its associated complications.

## 1 Introduction

Chronic Myeloid Leukemia (CML) is a malignant proliferative hematopoietic disease, a type of cancer in bone marrow. One of the hallmarks of CML is the unchecked proliferation of myeloid cells, a subset of white blood cells. CML causes the rise of white blood cell counts in the blood. In general, CML affects elder people and rarely occurs in children. Diagnosing CML at its later stage of the blastic phase helps to treat the disease and increase the patient's life span [1]. In most cases, CML does not cause any specific symptoms, it causes common symptoms such as pain in the bone, fever, weight loss, loss of appetite, fatigue, easy sweating, and blurry version. Various biomarkers helping to identify and diagnose a wide range of diseases and aid to read out the changes in the cells and organs in the body [[2], [3], [4], [5], [6], [7], [8], [9], [10], [11], [12]]. CD 19 antigen is the transmembrane glycoprotein belonging to the immunoglobulin superfamily, over-expressed in B cell lymphomas, B cell chronic lymphocytic leukemia, and B cell acute lymphocytic leukemia [13]. Therefore, CD 19 plays a major role as a therapeutic target for the treatment of leukemia and lymphoma and also recognized as a potential biomarker and target treatment for CML [14]. Apart from that, this protein is involved in various cell-related activities such as proliferation and B cell growth. This research is focused on developing a CD 19 biosensor on a single-walled carbon nanotube (SWCN) created with a circular-pattern of interdigitated electrode (IDE). An example of an electrochemical sensor is an IDE biosensor, which uses two electrodes on a substrate that are woven together. The electrodes are set up in a way called interdigitation, which resembles combs with one electrode's "fingers" interlocking with another electrode's fingers. Usually, conductive materials like carbon, platinum, or gold are placed on non-conductive substrates like silicon or glass to create electrodes. Impedance, capacitance, conductance, or current are examples of the observable electrical signals produced by the interaction of the target analyte with the functionalized electrode. Due to their high sensitivity, these sensors are frequently utilized to identify different biological substances.

Biosensors are widely employed in a variety of industries, such as biotechnology, environmental monitoring, food safety, and medical diagnostics [15,16]. A device that combines biological molecules and nanotechnology to identify and examine particular biological markers or chemicals is called a nanomaterial biosensor. Because of their special qualities at the nanoscale, nanomaterials are well-suited for biosensing applications. Although nanomaterial biosensors have a lot of potential, issues including repeatability, biocompatibility, and long-term stability must be resolved before they can be widely used in a variety of applications. Nanomaterials are nanosized materials that span across various industries, from biomedical and cosmetic to purification and environmental preservation. In particular, nanomaterials applications are widely accepted in various medical applications, such as biosensors, targeted-delivery, drug-screening, tissue-engineering, and various artificial bone-related applications [[17], [18], [19], [20], [21]]. Among the other metals carbon and its derivatives are predominantly used for various biomedical applications, in particular with high-performance biosensors. Due to its excellent features of adsorption, electrochemical performances, and mechanical flexibility and strength, it serves as an attractive material for biosensors. SWCN is a carbon-derived material having a higher surface area, strong mechanical strength, and good transport capabilities, which helps to develop an improving biosensor. In this study, a circular-pattern of IDE was modified into SWCN through the amine-linker and COOH-ended aptamer was attached on the SWCN to detect the level of CD19 antigen.

Aptamers, termed artificial antibodies, are selected from the method called SELEX. Aptamers are functionally comparable with antibodies with advantages such as, smaller in size, easy chemical modification, easy production, higher stability, lesser immunogenicity, and high binding affinity with the target [[22], [23], [24]]. In addition, the dissociation constant of aptamers with targets is in the range of nanomolar to picomolar. Aptamers could be applied instead of antibodies for therapeutic, and diagnostic applications in various diseases including cancer, and autoimmune diseases [[25], [26], [27]]. Aptamers are conjugated with nanomaterials and attached on the sensing electrode to enhance the analytical performances. Herein, the aptamer selected against CD 19 antigen was used as the probe molecule to detect the CD 19. Aptamers were immobilized on the IDE through the amine-modified SWCN. SWCN increases the number of aptamer immobilization on IDE and attracts a higher number of CD 19 and lowers the detection limit of CD 19. Further, to suppress the biofouling PEG-COOH was used as the blocking agent on the aptamer-modified surfaces and improved the CD 19 interactive analysis.

## 2 Materials and methods

### 2.1 Reagents and biomolecules

(3-Aminopropyl)trimethoxysilane (APTMS), Phosphate buffer saline (PBS), Potassium hydroxide (KOH), Recombinant human CD19 protein, PEG-COOH were ordered from Sigma Aldrich (USA). The single-walled carbon nanotube (SWCN) was purchased from Shenzhen Nanotech Port Ltd. Co. (Shenzhen, China). The following aptamers with 6-carbon spacer were commercially synthesized and received from a supplier (Apical Scientific, Malaysia) [28], [specific aptamer sequence: 5′-C6-COOH-TGCGTGTGTAGTGTGTCTGTTCTCCTTTTTTTGGTTGCTGCTCTTA GGGATTTGGGCGG-3′ and complementary aptamer sequence: 5′-C6-COOH-

ACGCACACATCACACAGACAAGAGGAAAAAAACCAACGACGAGAATCCCTAAACCCGCC-3’]. All measurements were done at 0–2 V with the step at 0.1 V on a wet surface under room temperature. Thorough washings were done with 10 reaction volumes of 10 mM PBS (pH 7.4), in between each step.

### 2.2 Construction of aptasensor on SWCN-modified IDE

IDE was designed with AutoCAD software and contain the dimensions: 10000 μm in length, 5000 μm in diameter, two 1500 μm probing sites, and 16 pairs of 4.5 μm thick finger and gap areas. Aptasensor was constructed on SWCN-modified IDE. For this process, aptamer was immobilized on IDE through the amine-modified SWCN. The following steps are carried out to attach the aptamers to the sensing electrode. (i) IDE was tethered by 1 % of KOH for 10 min and removed the excess KOH by rinsing with distilled water; (ii) SWCN (1 g/mL; dispersed in APTMS) was added and rested for 2 h and then washed the electrode with diluted EtOH; (iii) COOH-aptamer (1 μM) was dropped and waited for 1 h and washed the excess aptamer by 10 mM PBS (pH 7.4); (iv) PEG-COOH (1 mg/mL) diluted in PBS was introduced for 30 min and then rinsed the surface. The current-volt graph was recorded for each immobilization process to follow up the aptamer immobilization.

### 2.3 Quantification of CD 19 on aptamer attached IDE

CD 19 was quantified by aptamer, which was immobilized on IDE through the SWCN. Different concentrations of CD 19 were diluted (10 nM, 20 nM, 40 nM, 60 nM, 80 nM, and 100 nM) and dropped on SWCN-aptamer attached surfaces and rested for 30 min. After washing the surface with PBS, the current-volt measurement was recorded for each concentration of CD 19. The difference in current response was calculated (difference between before and after adding CD 19) for each concentration of CD 19 and plotted in a linear regression line to calculate the limit of detection of CD 19.

### 2.4 ELASA for CD 19 quantification

ELASA was constructed on SWCN-modified polystyrene plate (PS). For this process, aptamer was immobilized on the ELISA well through the amine-modified SWCN. The following steps were carried out to attach the aptamers to the ELISA well. (i) PS was tethered by 1 % of KOH for 10 min and removed the excess KOH by rinsing with distilled water; (ii) SWCN (1 g/mL; dispersed in APTMS) was added and rested for 2 h and then washed the well with diluted EtOH; (iii) COOH-aptamer (1 μM) was dropped and waited for 1 h and washed the excess aptamer by 10 mM PBS (pH 7.4); (iv) PEG-COOH (1 mg/mL) diluted in PBS was introduced for 30 min and then rinsed the surface; (v) Diluted CD19 (10–100 nM) was added and rested it for 30 min; (vi) Anti-CD-19 antibody (1:1000 dilution) was added; (vii) HRP conjugated secondary antibody was added. After 30 min wash the well with buffer and add the substrate for HRP to see the color changes.

### 2.5 Biofouling and serum spiking experiment on aptamer attached IDE

Negative controls with complementary aptamer and control proteins were performed to prove the biofouling effect on SWCN-aptamer-PEG–COOH–modified electrode surface. COOH-ended complementary aptamer was added on APTES-SWCN modified IDE and then CD 19 with the concentration of 100 nM was dropped. Current-volt measurement was recorded. Similarly, a control protein (CD 33 antigen) with a concentration of 100 nM was added to the SWCN-aptamer attached IDE, and the current-volt measurement was monitored. This current response was compared with the specific binding of CD 19 with its aptamer. Similarly selective detection of CD 19 was confirmed with the CD 19-spiked serum detection [diluted as 1:100 using 10 mM PBS (pH 7.4)]. on IDE. CD 19 concentrations from 10 to 100 nM were diluted in human serum and placed on an aptamer attached electrode surface and then the current responses were noted for each CD 19 to confirm the selective detection of CD 19 antigen.

## 3 Results and discussion

**Fig. 1:**
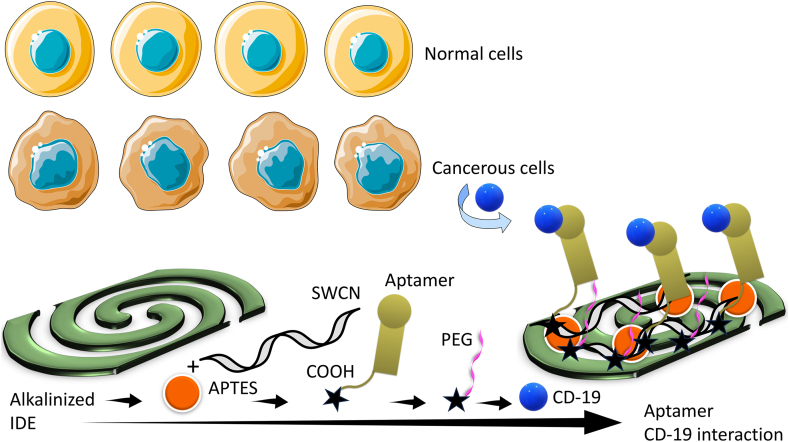
(a) Schematic for chronic myeloid leukemia IDE-sensor. IDE was tethered by KOH and amine-SWCN was functionalized. Subsequently, COOH ended aptamer was attached on SWCN. Further, the free amine surface was covered with PEG-COOH and finally, CD-19 antigen was interacted to detect the level of CD-19.

**Fig. 2:**
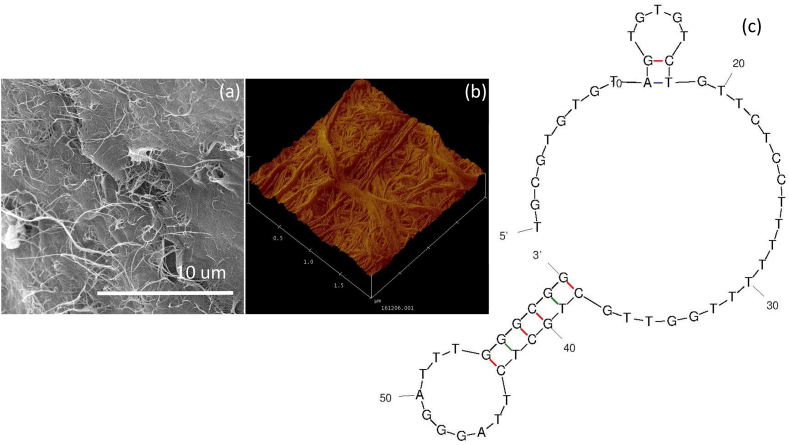
(a) SEM observation on SWCN; (b) AFM observation on SWCN; (c) Predicted secondary structure of aptamer sequence. The number of bases are indicated.

Chronic myeloid leukemia (CML) is a blood cancer that originates in the blood-forming myeloid cells in the bone marrow. Early identification of CML helps to provide a better treatment and increase the life-span. Various research proved that, after therapy, the condition of CML is from a life-threatening to a chronic illness and has a normal life span. Further, diagnosing CML with blood-based biomarkers is mandatory for diagnosing and monitoring the follow-up treatment. This research developed a highly sensitive CD19 antigen assay on SWCN-modified IDE for diagnosing CML. Fig. 1 shows the schematic representation for the CML sensor on IDE. As shown in the figure, IDE was treated with KOH to change the surface into hydroxylated (Fig. 1). After hydroxylation, APTES-SWCN was introduced on the IDE and then COOH-ended aptamer was attached on the SWCN through the amine linker. Further, the molecule-free IDE surface was covered with PEG-COOH and finally CD 19 antigen was placed to monitor the binding of CD 19 with its aptamer. In this study, SWCN was used to enhance the aptamer attachment on the electrode. In general, on the amine-modified surface, antibodies, aptamers, and proteins were immobilized through electrostatic or chemical interaction. However, this will lead to the unorganized and unstable immobilization of biomolecules, which reduces the chances of target-probe interaction. To overcome this issue, SWCN was coated with APTMS and attached to the IDE. On the surface of SWCN, a higher number of APTMS was attached, which attracts a higher number of COOH-ended aptamer. In addition, the attachment of SWCN on the sensing surface enhances the surface area with the ultimate capturing of desired molecules. This will increase the chance of improving the CD 19 antigen and aptamer interaction and lower the detection limit of CD 19. The sensing surface intactness was confirmed by atomic force microscopy (Fig. 2a and b) and the secondary structure of aptamer was predicted by mfold online software (Fig. 2c).

### 3.1 Aptasensor construction on IDE

**Fig. 3:**
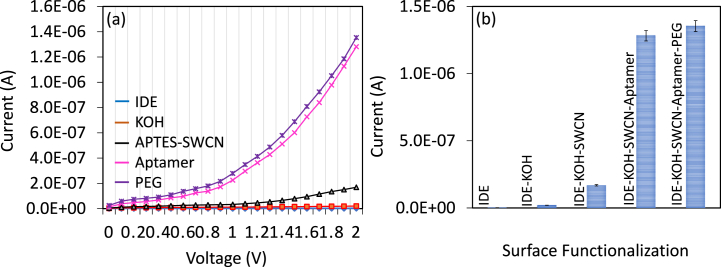
(a) Current-volt graph on aptamer immobilization. Clear current increment was recorded after the aptamer attachment on the electrode surface. (b) Current response of aptamer immobilization process. Aptamer displays the highest increment of the current response. Measurements were with the average of triplicates.

Aptamer-based sensor was constructed on SWCN-modified IDE. Aptamer was immobilized on IDE by using the amine-modified SWCN. Fig. 3a shows the current-volt graph of the aptamer functionalization process on IDE. Bare IDE recorded the current as 1.9 E−09 A and after IDE was hydroxylated with KOH, the current was increased to 10 2.03 E−08 A. Then amine-modified SWCN was added, current change was recorded as 1.69 E−07 A, which indicates the modification of IDE with SWCN through APTMS. This immobilization happened through KOH on the IDE and the functional group with the APTES. Further, when the COOH-ended aptamer was attached, it was noticed that drastically increasing the current to 1.28 E−06 A, this confirms the aptamer attachment on IDE through COOH in the aptamer and the amine on SWCN. Finally, PEG-COOH was added, and a little bit increment in current was noted due to the higher occupancy of aptamer on the amine-modified SWCN. A clear increment in current was recorded for each surface functionalization process (Fig. 3b), indicating the successful attachment of aptamers on IDE. This highly immobilized aptamer-modified IDE was utilized to detect the CD-19 antigen.

### 3.2 Determination of CD-19 antigen on SWCN-aptamer-modified IDE

**Fig. 4:**
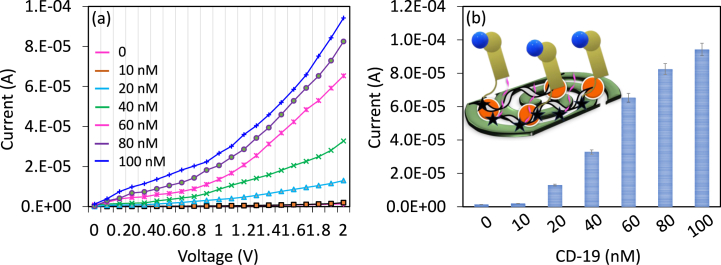
(a) CD 19 antigen determination. Current-volt graph of CD 19 antigen interactions with the immobilized aptamer. The current level was increased by increasing CD-19 concentration. (b) The current response of CD 19 detection. From 20 nM of CD 19, a clear difference in current responses was noted and from 60 nM, the current slowly decreased towards the saturation point. Figure inset shows the diagrammatic representation. Measurements were with the average of triplicates.

**Fig. 5:**
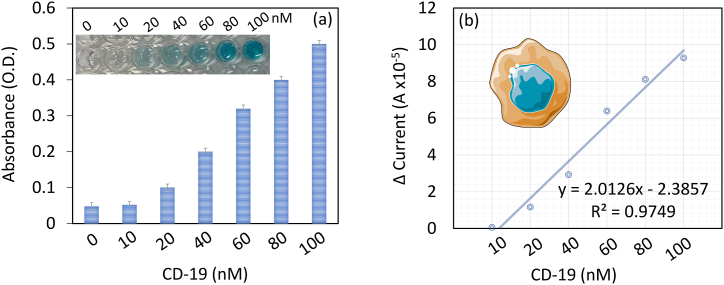
(a) ELASA for CD 19 quantification. With zero and 10 nM there is no increment in absorbance noted. From 20 nM of CD 19, clear increment of absorbance were noted. (b) Limit of detection of CD 19. The difference of current for each CD 19 was plotted in an Excel spreadsheet and found as the detection limit of CD 19 to 10 nM with the R^2^ value of 0.9749.

CD 19 antigen was determined on SWCN-aptamer modified IDE. Different concentrations of CD-19 (10–100 nM) were dropped on aptamer attached IDE and the changes in current were recorded. Fig. 4a displays the current-volt graph of CD 19 antigen interaction with the immobilized aptamer. As shown in the figure with zero CD 19 antigen (PEG-COOH blocked IDE), the surface shows the level of current as 1.35 E−06 A, after dropping of 10 nM of CD 19, the current was slightly increased to 1.95 E−06 A, which was due to the binding of CD 19 with its aptamer. Further, by increasing the CD 19 antigen concentration to 20, 40, 60, 80, and 100 nM, the current responses were improved to 1.3 E−05 A, 3.28 E−05 A, 6.53 E−05 A, 8.25 E−05 A, and 9.42 E−05 A, respectively. It was noted that from 20 nM of CD 19, the surface shows a clear difference in current responses and from 60 nM, the response was progressed slowly towards the saturation point (Fig. 4b). Similar surface modifications were conducted on the ELISA well to quantify the level of CD19. At 0 nM and 10 nM CD19, no absorbance changes were observed. With increasing concentrations of CD19, the absorbance also increased, and the color changes were clearly noticed (Fig. 5a)**.** The difference in current for each CD-19 concentration (from without CD 19) was plotted in an Excel spreadsheet and found as the detection limit of CD 19 as 10 nM with an R^2^ value of 0.9749 (Fig. 5b).

### 3.3 Biofouling and selectivity experiments on IDE

**Fig. 6:**
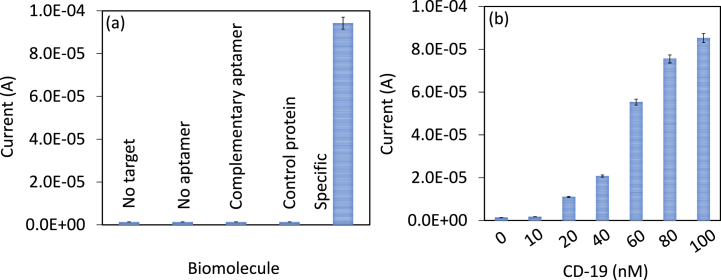
(a) Biofouling experiment for specific CD 19 detection. Experiments with control protein and complementary aptamer did not increase the response of current, confirming the specific recognition of CD 19 antigen. Measurements were with the average of triplicates. (b) Selective detection of CD 19. CD19 concentrations from 10 to 100 nM were diluted in human serum and detected by its aptamer. The current level was gradually increased by increasing the concentrations of CD 19, indicating selective CD 19 detection without interference. Measurements were with the average of triplicates.

Reducing biofouling is mandatory to improve the performance of biosensor. Higher biofouling attachment on the sensing electrode surface leads to a false-positive result. In this work, amine modification with APTMS was followed to attach SWCN and aptamer on the sensing electrode, which easily attracts the biomolecules and blocks the specific interaction. To avoid this situation, we used PEG-COOH to cover the excess APTES on the SWCN, which helps to interact CD 19 on the sensing surface specifically to aptamer. PEG-based polymer for surface functionalization helps to improve the biosensing and analytical performances. It was proved that PEG provides the stability of the biomolecules and gives excellent orientation on the sensing surface for lowering the detection limit. To prove the biofouling effect of the sensing electrode, control experiments with complementary aptamer, and control protein (CD 33) were tested on IDE. As shown in Fig. 6a, control experiments did not increase the response of current, confirming that there is no false positive current response was recorded and the sensing electrode surface only specifically recognizes the CD 19 antigen. Similarly, selective detection of CD-19 was confirmed with the CD 19-spiked serum detection on IDE. CD 19 concentrations from 10 to 100 nM were spiked into human serum and dropped independently on aptamer-modified IDE and then the current response was recorded for each CD 19 concentration. As shown in Fig. 6b, the current levels were gradually increased with increasing concentrations of CD 19, indicating selective CD 19 detection without interference.

## 4 Conclusion

Chronic myeloid leukemia (CML) is a cancer that originates in the bone marrow caused by the proliferation of granulocyte cells at all the maturation stages. Early identification, before the blastic phase is mandatory to increase the patient survival rate. This research generated an aptamer interdigitated electrode (IDE) sensor for CD 19 detection, which is a suitable biomarker for CML. A higher number of aptamer immobilization was achieved through the amine-modified SWCN, which identifies the CD 19 as low as 10 nM. Furthermore, biofouling experiments with control proteins and complementary aptamer did not show any increment in current responses, and also CD-19-spiked serum shows a clear enhancement of current responses confirming the selective and specific detection of CD 19. This SWCN-aptamer-modified IDE identifies CD 19 and helps to diagnose CML and its complications. The generated sensing platform in this study is a common and well-suited for other cancer markers. Even though, this sensing system improves the performance, the selection of probe and target with a high interaction is critical. Further, usage of different surface physical modifications with different nanomaterials will provide different insights.

## CRediT authorship contribution statement

**Chuntao Zong:** Writing – review & editing, Writing – original draft, Investigation, Formal analysis, Data curation. **Xuebing Ran:** Writing – review & editing, Visualization, Validation, Supervision, Resources, Project administration, Methodology, Investigation, Funding acquisition, Conceptualization.

## Declaration of competing interest

The authors declare that they have no known competing financial interests or personal relationships that could have appeared to influence the work reported in this paper.
